# NO-Donating NSAIDs, PPAR*δ*, and Cancer: Does PPAR*δ* Contribute to Colon Carcinogenesis?

**DOI:** 10.1155/2008/919572

**Published:** 2008-05-29

**Authors:** Gerardo G. Mackenzie, Shaheen Rasheed, William Wertheim, Basil Rigas

**Affiliations:** ^1^Division of Cancer Prevention, Department of Medicine, Stony Brook University, Stony Brook, NY 11794-5200, USA; ^2^Division of Gastroenterology, Department of Medicine, Stony Brook University, Stony Brook, NY 11794-5200, USA; ^3^Division of General Medicine, Department of Medicine, Stony Brook University, Stony Brook, NY 11794-5200, USA

## Abstract

The chemopreventive NO-donating NSAIDs (NO-NSAIDs; NSAIDs with an NO-releasing moiety) modulate PPAR*δ* and offer the opportunity to revisit the controversial role of PPAR*δ* in carcinogenesis (several papers report that PPAR*δ* either promotes or inhibits cancer). This review summarizes the pharmacology of NO-NSAIDs, PPAR*δ* cancer biology, and the relationship between the two. In particular, a study of the chemopreventive effect of two isomers of NO-aspirin on intestinal neoplasia in *Min* mice showed that, compared to wild-type controls, PPAR*δ* is overexpressed in the intestinal mucosa of *Min* mice; PPAR*δ* responds to *m*- and *p*-NO-ASA proportionally to their antitumor effect (*p*- > *m*-). This effect is accompanied by the induction of epithelial cell death, which correlates with the antineoplastic effect of NO-aspirin; and NO-aspirin's effect on PPAR*δ* is specific (no changes in PPAR*α* or PPAR*γ*). Although these data support the notion that PPAR*δ* promotes intestinal carcinogenesis and its inhibition could be therapeutically useful, more work is needed before a firm conclusion is reached.

## 1. INTRODUCTION

Cancer represents a major health challenge of our time. In the last decade, biomedical science has pursued with unusual vigor the molecular understanding of cancer. Cell signaling cascades have, in particular, been examined or even recognized in the context of cancer research. The implicit assumption (as well as the expectation) has been that understanding the mechanisms of carcinogenesis will facilitate the development of rational, mechanism-driven interventions for either the treatment or even better the prevention of cancer. The ultimate “deliverable” of such systematic efforts will be successful cancer therapeutic or preventive agents.

As is, however, sometimes the case in science, mechanistic progress can also be made while trying to understand the mode of action of agents already developed. Such appears to be the case with the opportunity that presented itself while we were exploring the mode of action of a novel chemopreventive agent, nitric oxide-donating aspirin (NO-ASA), and its relationship to peroxisome proliferator-activated receptor *δ* (PPAR*δ*). Here, we discuss our findings, and to provide an appropriate perspective, we summarize relevant aspects of the pharmacology of nitric oxide-donating nonsteroidal anti-inflammatory drugs (NO-NSAIDs), PPAR*δ* cancer biology, and the relationship between the two.

## 2. NO-NSAIDs AND CANCER

**Figure 1:**
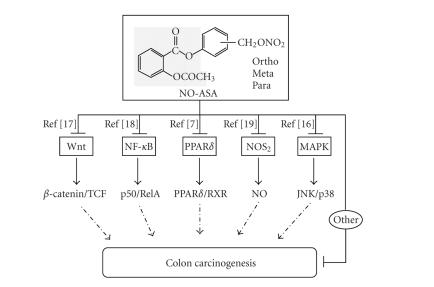
Effect of NO-ASA on PPAR*δ* and other signaling pathways. NO-ASA consists of a traditional ASA molecule (shaded), the spacer, and –ONO_2_, which releases NO, with the molecule being considered responsible for much of its pharmacological properties. There are three positional isomers of NO-ASA (*ortho*, *meta*, and *para*), depending on the position of –ONO_2_ in the benzene ring with respect to the ester bond linking the ASA and spacer moieties. NO-ASA affects several cell signaling pathways, all relevant to carcinogenesis. The modulation of these often cross-talking pathways culminates in a net inhibitory effect on cell growth, one of the crucial determinants of the fate of a tumor. It is likely that such mechanistic pleiotropism by NO-ASA is central to its efficacy against cancer.

NO-ASA, initially intended for rheumatologic and cardiovascular applications [1, 2], is a member of a large family of pharmacologically active compounds known as NO-donating NSAIDs (NO-NSAIDs). NO-NSAIDs consist of a conventional NSAID to which the NO-releasing moiety–ONO_2_ has been attached via a chemical linker [3, Figure 1]. In the case of NO-ASA, the spacer can vary in its chemical structure, generating a great number of derivatives. There are three positional isomers of the NO-ASA molecule (*ortho*, *meta*, and *para*), generated by varying the position of the –CH_2_ONO_2_ group with respect to the ester bond linking the two benzenes [4]. NO-ASA is the best studied NO-NSAID to date.

The impetus for the development of NO-NSAIDs for cancer prevention has been provided by extensive epidemiological data and interventional studies which over fifteen years have established conclusively conventional NSAIDs as chemopreventive agents against colon and other cancers [5, 6]. The limited efficacy (less than 50%) and side effects that accompany NSAIDs have prompted the search for better performing agents. NO-NSAIDs, especially NO-ASA, promise to be such an alternative, and their anticancer properties are now under intense study by our group and others. Even though significant progress has been made, the mechanism by which NO-ASA exerts its chemopreventive effect against colon cancer is still not completely understood [2]. Our data indicate that NO-ASA could exert its colon chemopreventive effect, at least in part, by modulating PPAR*δ* function [7].

Extensive preclinical results have established that NO-ASA, which is now FDA approved for clinical trials, displays properties consistent with a chemopreventive effect [8]. These findings can be grouped into those documenting a favorable in vitro cytokinetic effect and those demonstrating chemopreventive efficacy in animal models of cancer. Compared to their corresponding parent compounds, several NO-NSAIDs (including NO-ASA, NO-sulindac and NO-ibuprofen, NO-salicylic acid, NO-indomethacin, and NO-flurbiprofen) have greater potency in inhibiting the growth of cancer cell lines, for example, colon, prostate, lung, pancreas, tonsil, breast cancer, and leukemia [9–12]. For example, in the case of colon cancer cell lines, the IC_50_ values of NO-NSAIDs were enhanced between 1.7- and 1083-fold. The growth-inhibitory effect of NO-NSAIDs is due to a profound cytokinetic effect, consisting of reduced cell proliferation, enhanced cell death, and inhibition of cell-cycle-phase transitions. Beyond classical apoptosis, NO-ASA induced another form of cell death, termed as atypical cell death [4]. Likely, a form of cell necrosis, atypical cell death, was initially described in vitro, but may actually occur in vivo [7].

The in vivo studies used orthotopic animal models of cancer as well as xenotransplants of human cancer cell lines in appropriate murine hosts. For colon cancer, the results from the various models are congruent and demonstrate a clear-cut chemopreventive effect. In *Min* mice, 3 weeks of treatment with NO-ASA decreased the number of tumors by 55% [13]. In F344 rats treated with the carcinogen azoxymethane, NO-indomethacin and *meta* NO-ASA significantly suppressed both tumor incidence and multiplicity (NO-indomethacin was more effective than NO-ASA). Of the two NO-ASA isomers, the *para* was more efficacious than the *meta* in *Min* mice [4]. When combined with 5-fluorouracil or oxaliplatin, *para* NO-ASA showed additive effects [14]. Sequential NO-ASA and oxaliplatin treatment reduced tumor growth more effectively than single-drug treatments, perhaps by sensitizing colon cancer cells to the effect of antitumor drugs. Studies using a hamster model of pancreatic cancer generated impressive results [15]. Compared with the control group, NO-ASA reduced the incidence and multiplicity of pancreatic cancer by 88.9% and 94%, respectively, whereas conventional ASA had no significant effect.

An exciting aspect of NO-ASA is its extraordinarily enhanced potency. We and others have attempted to understand this through studies assessing their effects on potentially informative pathways (summarized in [8]). It appears that NO-ASA has a pleiotropic effect involving several pathways, as depicted in Figure 1. PPAR*δ* is a significant component of this array of signaling molecules [7, 16–19]. Below, after an overview of the role of PPAR*δ* in cancer, we discuss the relationship between NO-ASA and PPAR*δ*.

## 3. PPAR*δ* AND CANCER

PPARs, having their first member cloned in 1990 [20], are ligand-activated transcription factors belonging to the superfamily of nuclear receptors. They facilitate the response of cells to extracellular stimuli by transcriptionally regulating gene expression [21, 22]. Three distinct PPAR subclasses have been identified: PPAR*α*, PPAR*δ* (also referred to as PPAR*β*/*δ*), and PPAR*γ*. These isoforms are encoded by separate genes and differ in their tissue distribution and function. PPAR*δ* is the more ubiquitously expressed isoform. Each of the PPAR isoforms heterodimerizes with the 9-*cis*-retinoic receptor, their obligate partner. PPARs regulate diverse physiological processes ranging from lipogenesis to inflammation, and have been implicated in several disorders including the metabolic syndrome, diabetes, and atherosclerosis, as well as cancer. More recently, PPAR*γ* was shown to play a significant role in cell growth, inflammation, apoptosis, and angiogenesis [23–27].

The study of PPAR*δ* lags behind our fairly advanced understanding of PPAR*α* and PPAR*γ*; the development of high-affinity PPAR*δ* agonists has recently expedited progress [28, 29]. PGI_2_ and cPGI are naturally occurring PPAR*δ* agonists [30]. PPAR*δ* is involved in a wide range of phenomena affecting several functions, and some of them are critical to the life of an organism. PPAR*δ* stimulates fatty acid oxidation in heart and skeletal muscle [31, 32], and plays a role in cell differentiation [33–35], placental development [36], cancer, wound repair [37], and atherosclerosis [38–41]. PPAR*δ*-null mouse models revealed that PPAR*δ* deficiency is associated with multiple developmental and metabolic abnormalities, including frequent embryonic lethality [36].

## 4. THE PROS AND CONS FOR A ROLE OF PPAR*δ* IN CANCER

There have been both significant work on and significant excitement about a potential role of PPAR*δ* in cancer. As with any evolving field, some controversy is almost inevitable. This controversy arises mainly from the varying results from animal studies (summarized in [42]). Available data can be divided into two: those which support the notion that PPAR*δ* plays a crucial role in carcinogenesis, and those which indicate that PPAR*δ* is devoid of any such role. Below, we present the main points supporting each one of these antithetic conclusions (Table 1).

### 4.1. Pros

PPAR*δ* was ascribed as an oncogenic function after being identified as a direct transcriptional target of *β*-catenin, and as a repression target of the NSAID sulindac, a potent suppressor of colorectal tumors [44]. A close association between PPAR*δ* and colon carcinogenesis was suggested by immunohistochemical analyses showing that the expression of PPAR*δ* increases progressively as the colonic epithelium advances from normal to malignant [45].

A series of observations in *Min* mice support a procarcinogenic role of PPAR*δ*. When *Min* mice were treated with azoxymethane, PPAR*δ* levels were increased in flat dysplastic aberrant crypt foci [65], although the same authors indicate that PPAR*δ* expression in adenomas from *Min* mice does not differ compared to normal epithelium [65]. Deletion of PPAR*δ* decreased intestinal adenoma growth and inhibited the tumor-promoting effects of a PPAR*δ* agonist [51]. Interestingly, the same group also showed that prostaglandin E_2_ (PGE_2_), the predominant prostanoid found in most colorectal cancers, indirectly transactivates PPAR*δ* promoting cell survival and intestinal adenoma formation [53]. PGE_2_ treatment did not increase intestinal adenoma burden in *Min* mice lacking PPAR*δ*, concluding that PPAR*δ* is a focal point of cross-talk between the prostaglandin and Wnt signaling pathways, which results in a shift from cell death to cell survival, leading to increased tumor growth [53]. Treatment of *Min* mice with a synthetic agonist of PPAR*δ* increased significantly the number and size of intestinal polyps. The most prominent effect was on polyp size; the PPAR*δ* activator increased the number of polyps by >2 mm five-fold [47]. The same group also showed that compared with control *Apc* ^Min/+^ mice (*Ppard* ^+/+^/*Apc* ^Min/+^), small intestinal polyps in PPAR*δ*-deficient *Apc* ^Min/+^ mice (*Ppard* ^−/−^/*Apc* ^Min/+^) were reduced three-fold; the number of large polyps (>1 mm) was reduced about ten-fold. Heterozygous deletion of PPAR*δ* (*Ppard* ^+/−^/*Apc* ^Min/+^) did not significantly reduce the total number of small and large intestinal polyps in malemice, but this disruption significantly diminished the number of small intestinal polyps that were >1 mm [51].

In cultured colon cancer cells, PPAR*δ* inhibited differentiation, conferred apoptotic resistance, and promoted cell migration [43], whereas prostacyclin, a metabolic product of COX-2 which modulates intestinal tumorigenesis [66], increased PPAR*δ* activity [43]. PPAR*δ* expression was elevated in colon cancer cells and was repressed by *apc* via the *β*-catenin/TCF-4 response elements in its promoter [44]. Genetic disruption of *Ppard* decreased the tumorigenicity of human coloncancer cells [46]. HCT116 *Ppard* ^−/−^ cells, inoculated as xenografts onto nude mice, exhibited decreased ability to form tumors compared to *Ppard* ^+/−^ and wild-type controls [46]. Dietary fish oil/pectin protected rats against radiation-enhanced colon cancer by upregulating apoptosis in colonic mucosa, in part, by suppressing PPAR*δ* [48].

Data from noncolonic cell lines and tissues also support a role for PPAR*δ* in cancer. Activation of PPAR*δ* results in increased growth in sex hormone-responsive breast (T47D, MCF7) and prostate (LNCaP, PNT1A) cell lines [52]. Epithelial ovarian cancer cells express high levels of PPAR*δ*, and inhibition of PPAR*δ* reduced tumor growth [50]. In epithelial ovarian cancer cells, aspirin suppressed PPAR*δ* function and cell growth by inhibiting ERK1/2 [50]. Activation of PPAR*δ* by its pharmacologic ligand GW501516 enhanced the growth of human hepatoma cell lines, whereas PPAR*δ* knockdown by siRNA prevented cell growth [67]. In murine knockout experiments, targeted removal of a hub node (PPAR*δ*) of the angiogenic network markedly impaired angiogenesis and tumor growth [49]. In human cholangiocarcinoma, a positive feedback loop between PPAR*δ* and PGE_2_ was recognized; this interaction plays an important role in cell growth [68]. In patients with pancreatic cancer, PPAR*δ* levels were correlated with advanced pathological tumor stage, increasing the risk of tumor recurrence and distant metastases [49].

### 4.2. Cons

The strongest evidence that PPAR*δ* plays no appreciable role in carcinogenesis comes from a series of animal studies, cell culture data, and from studies evaluating the role of PPAR*δ* in inflammation, the latter being considered as a contributor to carcinogenesis.

Barak et al. evaluated the hypothesis that if PPAR*δ* is a critical transducer of the tumorigenic signal, then its loss should substantially reduce, if not eliminate, intestinal polyps in *Min* mice [36]. *Min* mice that were *Ppard*-null harbored intestinal and colonic polyps. Histologically, all of the 12 intestinal polyps from *Ppard* ^+/+^ mice and the 9 from *Ppard* ^−/−^ mice were low-grade tubular adenomas. The number of intestinal polyps was not significantly different between *Ppard* ^+/+^, *Ppard* ^+/−^, and *Ppard* ^−/−^ *Min* mice. Loss of PPAR*δ* did not significantly change the median size of intestinal polyps, although polyps > 1 mm were decreased upon PPAR*δ* dosage reduction, which was further pronounced for polyps > 2 mm. The number of polyps < 1 mm was essentially identical in all PPAR*δ* genotype groups. Their conclusion was that PPAR*δ* is qualitatively dispensable for the tumorigenic process, although they could not rule out the possibility that it influences the pace of polyp growth. In agreement with these findings, Marin et al. showed that PPAR*δ* activation by GW0742 inhibits colon polyp multiplicity in *Ppard* ^+/+^ but not in *Ppard* ^−/−^ mice, suggesting that ligand activation of PPAR*δ* attenuates azoxymethane-induced colon carcinogenesis [64].

The most striking result is provided by a study demonstrating that in *Min* mice differing in their *Ppard* genotype (*Ppard* ^−/−^, which did not express PPAR*δ* protein; *Ppard* ^+/−^; and *Ppard* ^+/+^), the incidence of polyp formation was not significantly different between groups [54]. In fact, *Ppard* ^−/−^ *Min* mice had about 3–6 times as many colon polyps as those of *Ppard* ^+/+^ or *Ppard* ^+/−^ mice. No significant differences in polyp size were found between any of the genotypes. Congruent results were obtained when they examined colon carcinogenesis with a more colon-specific, azoxymethane-induced model. The data from these two different colorectal cancer models suggest that PPAR*δ* attenuates colon carcinogenesis.

Finally, Reed et al. reported that PPAR*δ*-null *Min* mice exhibited increased predisposition to intestinal tumorigenesis [55]. Another report from the same group, evaluating the incidence and severity of intestinal neoplasia in mice deficient in both PPAR*δ* and the mismatch repair gene *Mlh1*, showed that deficiency of PPAR*δ* in mice with compromised mismatch DNA repair failed to affect intestinal neoplasia [59], with the implication being that PPAR*δ* is not required for intestinal adenoma formation.

Similar results have been obtained for noncolonic tumors. For example, mice lacking one or both alleles of *Ppard* had enhanced growth of lung tumors [58]. In another example, the onset of tumor formation, tumor size, and tumor multiplicity of the skin was significantly enhanced in PPAR*δ*-null mice compared with wild-type mice [69].

There are also data from cell culture models contradicting the notion that PPAR*δ* plays a role in carcinogenesis. For example, in several human cancer cell lines, two PPAR*δ* ligands failed to increase cell growth, Akt phosphorylation, or the expression of VEGF or COX-2 [56]. PPAR*δ* activation by a PPAR*δ* agonist does not induce cell growth in HT29, SW480, and HCA-7 colon cancer cells [64]. Furthermore, Raf oncogenes can contribute to tumorigenesis by augmenting the secretion of tumor growth promoting prostaglandins, such as PGI_2_. However, using several cell lines, Fauti et al. showed that the increase in PGI_2_ synthesis did not induce the transcriptional activity of PPAR*δ*, suggesting that the oncogenic effect of PGE_2_ does not involve PPAR*δ* [70]. Another PPAR*δ* function is the modulation of cell cycle. Knockdown of the PPAR*δ* gene by siRNA promoted proliferation of HCT116 cells, suggesting that PPAR*δ* may, in fact, inhibit their proliferation by arresting them in the G_1_ phase of the cell cycle [57].

The chemopreventive action of PPAR*δ* is also suggested by studies showing that in many cell types PPAR*δ* promotes differentiation and inhibits proliferation [33, 60, 69, 71]. For example, Hollingshead et al. examined in azoxymethane-treated PPAR*δ*-null mice whether PPAR*δ* activation and COX2 inhibition attenuate colon cancer independently. Inhibition of COX2 by nimesulide attenuated colon cancer, and activation of PPAR*δ* by GW0742 had inhibitory effects. The effects of these compounds occurred through independent mechanisms as increased levels of differentiation markers resulting from ligand activation of PPAR*δ* were not found with COX-2 inhibition, and reduced PGE_2_ levels resulting from COX-2 inhibition were not observed in response to ligand activation of PPAR*δ* [62]. In another study by the same group, wild-type (*Ppard* ^+/+^) and *Ppard* ^−/−^ mice were treated with azoxymethane, together with GW0742, a specific PPAR*δ* ligand, to test if *Ppard* ^−/−^ mice exhibit increased colon polyp multiplicity [64]. Ligand activation of PPAR*δ* in *Ppard* ^+/+^ mice increased the expression of mRNA encoding the adipocyte differentiation-related protein, fatty acid-binding protein, and cathepsin E, all being indicative of colonocyte differentiation [64]. Thus, the induction of differentiation and the inhibition of proliferation in response to PPAR*δ* activation support the hypothesis that PPAR*δ* attenuates colon carcinogenesis [62].

Another contrarian point of view concerns the role of PPAR*δ* in inflammation, with studies suggesting that activation of PPAR*δ* has anti-inflammatory effects. In hepatocytes, the PPAR*δ* agonist suppressed IL-6-mediated acute phase reaction, prompting the speculation that PPAR*δ* agonists may be used to suppress systemic inflammatory reactions in which IL-6 plays a central role [72]. Two synthetic PPAR*δ* ligands inhibited TNF*α*-induced expression of the vascular cell adhesion molecule-1 and E-selectin in human umbilical vein endothelial cells, suggesting that PPAR*δ* activation has a potent anti-inflammatory effect [61].

Relevant to cancer is the presumed role of PPAR*δ* in inflammation and NF-*κ*B regulation [63, 73, 74]. Such a role is exemplified by studies on the skin, where activation of PPAR*δ* by IFN-*γ* and TNF*α* accelerated keratinocyte differentiation [75]. Studies with PPAR*δ* agonists have shown anti-inflammatory properties of PPAR*δ* attributed to inhibition of NF-*κ*B DNA-binding activity [74, 76]. Inflammation induced by TPA (*O*-tetradecanoylphorbol-13-acetate) in the skin was lower in wild-type mice fed sulindac than in similarly treated PPAR*δ*-null mice [77]. In human endothelial cells, PPAR*δ* activators inhibited TNF*α*-induced endothelial inflammation (VCAM-1 expression, monocyte adhesion, and MCP-1 secretion), in part by interfering with the NF-*κ*B signaling pathway [63]. Lipopolysaccharide-induced TNF*α* production in cultured cardiomyocytes through NF-*κ*B activation was inhibited by overexpression of PPAR*δ* or the PPAR*δ* synthetic ligand GW0742 [73].

The foregoing arguments and counterarguments make it clear that this controversy remains unresolved. This is the reason why we attempted to obtain an insight into the role of PPAR*δ* in carcinogenesis by exploiting the unique opportunity offered by studying the effect of NO-ASA on PPAR*δ*. Our work is presented in the following section.

## 5. NO-NSAIDs AND PPAR*δ*

Our limited understanding of the mechanism by which NO-ASA exerts its colon chemopreventive effect combined with the possibility that PPAR*δ* plays a role in colon carcinogenesis prompted us to assess the expression of PPAR*δ* during intestinal carcinogenesis, and also whether NO-ASA modulates it [7].

We studied *Min* mice and their congenic (wild-type) mice, C57BL/6J^+/+^. Three groups of each type of mice were treated for 21 days with vehicle or *m*-NO-ASA or *p*-NO-ASA, each at 100 mg/kg/day. As expected from their relative in vitro potency, after 21 days *m*-NO-ASA suppressed the number of intestinal tumors in *Min* mice (wt mice had no tumors) by 38%, and *p*-NO-ASA by 59%.

Most of the PPAR*δ* positive cells (staining being always nuclear) were in the intestinal villi, with only few in the crypts. PPAR*δ* was minimally expressed among the three groups of wild-typemice. In contrast, the expression of PPAR*δ* in *Min* mice, similar in tumors and histologically normal mucosa, was more than ten-fold increased compared to wild-type mice. The two NO-ASA positional isomers inhibited the expression of PPAR*δ* in both normal and neoplastic cells of *Min* mice. *m*-NO-ASA suppressed PPAR*δ* expression in histologically normal mucosa by 23% and in neoplastic tissue by 41%; *p*-NO-ASA suppressed PPAR*δ* expression in histologically normal mucosa by 27% and by 55% in neoplastic tissue. The reduction in the number of tumors by each NO-ASA isomer and the respective suppression of PPAR*δ* expression in neoplastic cells are strikingly similar; the *meta* isomer reduced tumor incidence by 38% and PPAR*δ* expression by 42%, whereas the corresponding reduction for the *para* isomer was 59% and 55%. Of note, the expression of PPAR*α* and PPAR*γ* was sparse, and treatment with NO-ASA had no appreciable effect on either of them.

The changes in PPAR*δ* expression induced by NO-ASA seemed to have a significant impact on the cell kinetics of the intestinal mucosa, rendering such an effect mechanistically important. The induction of apoptosis by NO-ASA, more prominent in neoplastic epithelial cells, followed closely the pattern of PPAR*δ* reduction. Thus, in the neoplastic tissues, *m*-NO-ASA increased apoptosis by 22% and *p*-NO-ASA by 70%. The percentage of changes in PPAR*δ* expression and apoptosis is significantly correlated (*P* < .03), suggesting a potential etiological association between the two events.

**Figure 2:**
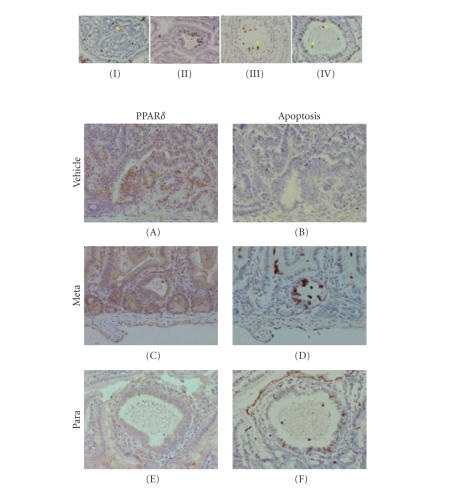
Effect of NO-ASA on PPAR*δ* and apoptosis in colon tissue of Min mice. Upper panel: the evolution of necrotic areas in intestinal tumors treated with *p*-NO-ASA; sections are stained by the TUNEL method. (I) Coalescence of TUNEL positive cells (arrow), representing the earliest stage; (II) abundant apoptotic cells at the margins of the developing area with contrast to their rarity in the surrounding area; (III) and (IV) the necrotic area is increasing in size, but TUNEL positive cells persist at its margins; TUNEL positive areas within the necrotic areas (arrows) suggest their cellular origin. *Lower panel*: the relationship of PPAR*δ* and apoptosis in NO-ASA treated intestinal tumors. Successive sections of intestinal tumors were stained for PPAR*δ* expression and apoptosis. The untreated tumor shows strong PPAR*δ* expression (A) and rare apoptotic cells (B). After treatment with *meta* or *para* NO-ASA, tumors show decreased PPAR*δ* expression (C) and (E) and increased apoptosis (D) and (F). Magnification is x400, adapted from Ouyang et al. [7].

We have previously reported that NO-ASA induces two types of cell death, classical apoptosis as well as atypical cell death, which based on a variety of criteria appears to be a variant of necrosis [4]. Documentation of atypical cell death in vivo had been elusive. This study, however, provided a glimpse into this phenomenon in vivo. As shown in Figure 2, we were able to record the evolution of necrotic areas in NO-ASA-treated intestinal tumors. Initially, TUNEL positive cells coalesce and, as the necrotic area develops, they populate its margins (being extremely rare in the surrounding tissue). As the necrotic area increases in size, the TUNEL positive cells persist at the margins. We have identified multiple TUNEL positive spots within the necrotic areas, suggesting their cellular origin. We believe that these TUNEL positive cells are necrotic cells [78]. The relationship of PPAR*δ* and cell death induced by NO-ASA was ascertained by studying successive sections of intestinal tumors from both treated and untreated animals (Figure 2). Untreated tumors show strong PPAR*δ* expression and few apoptotic cells. After treatment with *meta* or *para* NO-ASA, tumors show decreased PPAR*δ* expression and increased apoptosis. If the apoptosis index of tumors from NO-ASA-treated mice is plotted against the expression of PPAR*δ*, the association between the two is statistically significant; Figure 2 makes this correlation obvious. It should, however, be pointed out that these data have two methodological limitations. First, the specificity of the antibody is not considered by experts in the field ideal for immunohistochemistry, as nonspecific binding is possible. Second, no corroborating methodology was employed such as determination of PPAR*δ* protein levels in these tissues by immunoblotting.

Other NSAIDs such as aspirin (of which NO-ASA is a derivative) have been reported to have PPAR*δ* as one of their molecular targets. In epithelial ovarian cancer cells, aspirin suppressed PPAR*δ* function and cell growth by inhibiting ERK1/2 [50]. Sulindac sulfide and indomethacin inhibit both 14-3-3 proteins and PPAR*δ* levels in HT29 cells, suggesting that this could be the mechanism by which NSAIDs induce apoptosis in colorectal cancer [79]. Furthermore, in SW480 cells, sulindac sulfone significantly decreased PPAR*δ* expression more potently than the sulfide metabolite [80]. A case-control study in a large population showed that a polymorphism in the promoter of PPAR*δ* modified the protective effect of NSAIDs on colorectal adenomas [81]. However, the opposite was observed by another group, which found that regular NSAIDs use reduced the risk of colorectal cancer, but none of the polymorphic genes studied, including PPAR*δ*, modified their protective effect [82].

Several reports present evidence that NSAIDs induce apoptosis independently of PPAR*δ*. For example, sulindac significantly inhibited chemically induced skin carcinogenesis in both wild-type and PPAR*δ*-null mice [83]. In addition, aspirin-induced apoptosis in Jurkat cells was not mediated by PPAR*δ* [84]. Aspirin at a concentration which induces apoptosis did not affect the DNA binding of PPAR*δ*, whereas neither addition of a specific PPAR*δ* ligand nor transient transfection of PPAR*δ* expression vectors protected Jurkat cells from aspirin-induced apoptosis. Finally, as the work of Hollingshead et al. presented above suggested, COX-2 inhibition by the NSAID nimesulide and PPAR*δ* activation during colon carcinogenesis occurred through independent mechanisms [62].

## 6. CONCLUSIONS AND FUTURE DIRECTIONS

The contrast in data that were reviewed here on the potential role of PPAR*δ* in cancer, with colon cancer being most extensively evaluated, could not be starker. Excellent studies from fine laboratories led “conclusively” to diametrically opposite results. As no grey zone seems to exist, the reader is left in bewilderment.

Our data indicate that, compared to wild-type mice, the nuclear receptor PPAR*δ* is overexpressed in the intestinal mucosa of *Min* mice, and that two isomers of NO-ASA, which suppress their intestinal neoplasia, inhibit to a commensurate degree the expression of PPAR*δ* as well. This effect is accompanied by the induction of epithelial cell death, which correlates well with the antineoplastic effect of NO-ASA. As discussed earlier, these findings are, however, limited by the fact that PPAR*δ* was detected using an antibody whose specificity may not be perfect and also by the lack of any corroborating methodology (e.g., immunoblot detection of PPAR*δ* levels).

One could, nevertheless, consider that these findings support the notion that PPAR*δ* promotes colon carcinogenesis. The key elements of support come from three findings. First, PPAR*δ* is overexpressed in the intestinal mucosa of the *Min* mice but not in the wild-type control mice; being the same in histologically normal and neoplastic mucosas further suggests that it has a role in early events of carcinogenesis. There is also specificity in the induction of PPAR*δ*, as neither PPAR*α* nor PPAR*γ* was induced. Second, PPAR*δ* responds to two NO-ASA molecules that are structurally identical except for their positional isomerism, proportionally to their antitumor effect. And, third, changes in tumor response, PPAR*δ*, and cytokinetic parameters (apoptosis and necrosis) are closely correlated and mechanistically congruent.

Clarifying the role of PPAR*δ* in colon carcinogenesis and the response to medications is of substantial interest. The mechanistic significance of this question is apparent. The implications for the rational design of therapeutic and/or preventive approaches are also clear. Finally, the fact that PPAR*δ* agonists may be used for other indications raises the concern of unintended consequences of such modulation of PPAR*δ*, which may have a direct effect on the patient's risk of colon and perhaps other cancers.

At this stage, the jury should be considered out on the role of PPAR*δ* in cancer. As with any evolving field, the mundane but accurate conclusion is that more work is needed to clarify such an important question.

## Figures and Tables

**Table 1:** The *pros* and *cons* for a role of PPAR*δ* in cancer.

	Evidence	Reference
*Pros*		
	PPAR*δ* expression is enhanced in colon cancer cells	[43]
	PPAR*δ* expression is repressed by the APC gene	[44]
	PPAR*δ* expression increases as tumor progresses	[45]
	PPAR*δ* genetic disruption decreases tumorigenicity of colorectal cancer cells	[46]
	PPAR*δ* activation accelerates intestinal adenoma growth in *Min* mice	[47]
	*Ppard* ^−/−^ HCT116 cells exhibit decreased ability to form xenograft tumors	[46]
	Dietary fish oil/pectin protects against radiation-enhanced colon cancer by upregulating apoptosis, in part, through PPAR*δ* suppression	[48]
	PPAR*δ* expression levels are correlated with advanced pathological tumor stage in tumor patients	[49]
	PPAR*δ*-targeted removal of a hub node of the angiogenic network markedly impairs angiogenesis and tumor growth in mice	[49]
	Inhibition of PPAR*δ* function reduces growth of epithelial ovarian cancer	[50]
	Activation of PPAR*δ* upregulates VEGF in colon cancer cells	[51]
	PPAR*δ* activation stimulates the proliferation of human breast and prostate cancer cell lines	[52]
	PGE_2_ indirectly transactivates PPAR*δ* promoting cell survival and intestinal adenoma formation	[53]

*Cons*		
	PPAR*δ*-null *Min* mice exhibit increased predisposition to intestinal tumorigenesis	[54]
	PPAR*δ*-deficient mice show higher polyp formation	[55]
	PPAR*δ* agonists do not increase cell growth in human cancer cell lines	[56]
	PPAR*δ* is dispensable for polyp formation in the intestine and colon of *Min* mice	[36]
	RNA interference against *Ppard* promotes proliferation of HCT116 cells	[57]
	Lung tumorigenesis is attenuated in mice with disrupted *Ppard*	[58]
	PPAR*δ* does not modify impaired mismatch repair-induced neoplasia	[59]
	PPAR*δ* promotes differentiation, inhibiting cell proliferation in keratinocytes	[60]
	PPAR*δ* ligands inhibit TNF*α*-induced expression of the vascular cell adhesion molecule-1 and E-selectin in HUVEC, preventing inflammation	[61]
	Inhibition of colon carcinogenesis by a PPAR*δ* agonist in an azoxymethane mouse model	[62]
	PPAR*δ* activators inhibit TNF*α*-induced endothelial inflammation, in part by interfering with the NF-*κ*B signaling pathway	[63]
	PPAR*δ* activation by a PPAR*δ* agonist produces no change in colon cancer cell growth	[52]
	PPAR*δ* activation by GW0742 inhibits colon polyp multiplicity in *Ppard* ^+/+^ mice, but not in *Ppard* ^−/−^ mice	[64]

## ABBREVIATIONS

COX: Cyclooxygenase
MAPK: Mitogen-activated protein kinase
NSAIDs: Nonsteroidal anti-inflammatory drugs
NO-ASA: NO-donating aspirin
NO-NSAIDs: Nitric oxide-donating nonsteroidal anti-inflammatory drugs
PPAR: Peroxisome proliferator-activated receptor
TPA: *O*-tetradecanoylphorbol-13-acetate.
